# Detecting metastatic potential of cancer through longitudinal vasculature imaging of biomaterial scaffold using non-invasive *in vivo* photoacoustic microscopy and optical coherence tomography

**DOI:** 10.7150/thno.101685

**Published:** 2025-01-01

**Authors:** Zhanpeng Xu, Guillermo Escalona, Ian Schrack, Wei Zhang, Tianqu Zhai, Lonnie D. Shea, Xueding Wang

**Affiliations:** 1 Department of Biomedical Engineering, University of Michigan, Ann Arbor, Michigan, USA.; 2 Department of Radiology, University of Michigan, Ann Arbor, Michigan, USA.; 3 Department of Chemical Engineering, University of Michigan, Ann Arbor, Michigan, USA.

**Keywords:** Biomaterial scaffold, Breast cancer, Metastatic progression, Angiogenesis, Vascularization, Photoacoustic microscopy, Optical coherence tomography

## Abstract

Metastasis represents a stage in which the therapeutic objective changes from curing disease to prolonging survival, as detection typically occurs at advanced stages. Technologies for the early identification of disease would enable treatment at a lower disease burden and heterogeneity. Herein, we investigate the vascular dynamics within a synthetic metastatic niche as a potential marker of disease progression.

**Methods**: The synthetic metastatic niche consists of a biomaterial scaffold implanted subcutaneously, which supports the formation of a vascularized tissue that recruits immune cells due to the foreign body response that then leads to tumor cell recruitment. This defined site is analyzed with multi-modal imaging techniques, including photoacoustic microscopy (PAM) and optical coherence tomography (OCT), to monitor the changes in vasculature of the niche as a measure of metastatic progression. We investigated angiogenesis for three triple-negative breast cancer models (4T1, 4T07, and 67NR cell lines) with distinct metastatic capabilities.

**Results**: Longitudinal imaging with PAM and OCT offered high-resolution, 3D views of vascular morphology, revealing accelerated and disorganized vascular reorganization with metastases, in contrast to the stable vessels observed in the control and non-metastatic model. Quantitative image analysis of vascular parameters, such as vessel area density, vessel mean tortuosity, and total vessel length substantiated these observations, with significant differences in vascular metrics emerging as early as 8 days post tumor-inoculation in metastatic models.

**Conclusions**: This study identifies the potential for longitudinal monitoring of vascular remodeling at a subcutaneous site for assessing metastatic progression in triple-negative breast cancer.

## Introduction

Breast cancer is the most frequently diagnosed and the second most lethal cancer among women worldwide 1. Although the overall 5-year survival rate for breast cancer is 90%, this rate drops to just 28% in patients with advanced, metastatic disease 2. Metastases can invade distant organs like the lung or brain, impairing their function and leading to mortality 3. In particular, aggressive forms of breast cancer, such as triple negative breast cancer (TNBC), are often detected at later stages and have higher rates of mortality 4. For primary tumors, early detection leads to improved outcomes 5, due in part to less aggressive treatments and a lower risk of metastasis. Extending early detection from primary tumors to metastatic disease has the potential for similar benefits based on a lower disease burden and heterogeneity 6, yet the markers of early metastatic disease must be developed.

The current technologies for metastasis detection focus on identifying tumor cells within distant tissues, which often results in detection at a relatively late stage of disease. Imaging and tissue biopsy are performed after a patient self-reports symptoms, with symptoms of tissue dysfunction indicating a late stage of disease. Imaging is used to guide tissue biopsy, yet imaging is only able to identify lesions that are several millimeters in diameter, and biopsy cannot be routinely performed due to the risk associated with the procedure. Liquid biopsy, which typically measures circulating tumor DNA (ctDNA), has been proposed as an alternative method for disease surveillance. However, tumors only shed detectable levels of ctDNA when they have expanded significantly, so liquid biopsy detects disease at a stage comparable to imaging, offering no significant improvement towards the early detection of metastatic disease 7. We have proposed a microporous poly(ε-caprolactone) (PCL) scaffold as a synthetic metastatic niche due to its well-known biocompatibility and stability *in vivo*. PCL is used within multiple FDA approved medical applications, including surgical sutures 8, implantable composite meshes 9, and bone filler 10. The scaffold is generally considered biocompatible and is stable for several months, which supports its use for either imaging or tissue biopsy for longitudinal monitoring of disease progression. In a murine model of TNBC, these scaffolds were infiltrated by tumor cells, immune cells and stromal cells, providing a defined site for monitoring disease. Immune dynamics and gene expression in the native metastatic organ correlates with those in the scaffold 11. The detection of tumor cells directly in the scaffold is possible with biopsy, yet a less-invasive strategy may facilitate early disease detection based on the characteristics of the metastatic niche.

The vascular structures within a tissue are altered during cancer progression and have been denoted as a hallmark of cancer. In healthy tissues, a balance of pro- and anti-angiogenic factors is maintained, but cancer cells at metastatic sites exploit angiogenesis to meet their metabolic demands 12. Consequently, metastatic progression is associated with the secretion of angiogenic factors, leading to the formation of new, yet aberrant, vasculature that supplies nutrients and oxygen 13. This newly formed vasculature is typically immature, disorganized, hyperpermeable, and tortuous, which facilitates tumor cell intravasation, ultimately leading to colonization in distant organs and the formation of secondary tumors 14. The primary tumor influences these sites of secondary tumor formation by releasing factors like vascular endothelial growth factor (VEGF) into circulation, which alters the distant vasculature and allows access for immune and tumor cells 15, 16. Multiple studies have linked angiogenesis with metastasis, underscoring its significance in the growth of the primary tumor and throughout the metastatic process 17-19. However, limitations in imaging depth and the random nature of metastatic seeding have limited the utility of longitudinal imaging of vasculature *in vivo*.

In this report, we investigated imaging of the vasculature within the synthetic metastatic niche, with the goal of identifying quantitative changes that would correlate with metastatic progression. Photoacoustic imaging (PAI) offers a high-sensitive, label-free approach for monitoring the vasculature in the scaffold. As a hybrid technology, PAI combines the great sensitivity of optical imaging with the good tissue penetration capability of ultrasound imaging. PAI operates by irradiating tissue chromophores with short laser pulses, creating broadband ultrasound waves through optical absorption and thermal expansion, which are then captured to generate 3D images of the internal tissue structure 20, 21. Due to hemoglobin's high optical absorption of laser light, PAI is intrinsically sensitive in visualizing vasculature and monitoring its alterations 22. In particular, photoacoustic microscopy (PAM) offers high spatial resolution, enabling the detection of fine details in vascular structures, which is crucial for monitoring angiogenesis and the microvascular changes associated with tumor progression and metastasis. Previously, PAI has been successfully applied to characterize and analyze the glioblastoma at different stages 23, and to visualize early cancer hepatic micrometastasis label-free 24. In addition, as an optical scattering material, the scaffold can be imaged by using optical coherence tomography (OCT), depicting comprehensive microenvironment when combined with PAM results. Since the high penetration ability of near-infrared light, OCT can image through scattering media with high spatial resolution. In this study, we investigated the dynamic vasculature within the scaffold using three TNBC cell lines derived from the same BALB/c mammary tumor. These cell lines are commonly used in oncology to model varying degrees of aggressiveness 25-27: i) 67NR cells remain confined to the primary tumor, ii) 4T07 cells disseminate to distant tissues but do not form metastases, and iii) 4T1 cells are highly metastatic, particularly to the lung 28. Mice had scaffolds implanted and tumor cells were inoculated orthotopically, with a window placed above the scaffold to facilitate imaging. Longitudinal monitoring of vascular changes within the scaffold using PAM were able to distinguish between these metastatic models, demonstrating a correlation between the vascular structure and the metastatic cascade. The described method may ultimately provide a novel strategy for tracking cancer progression.

## Materials and Methods

### Imaging system setup

**Figure 1:**
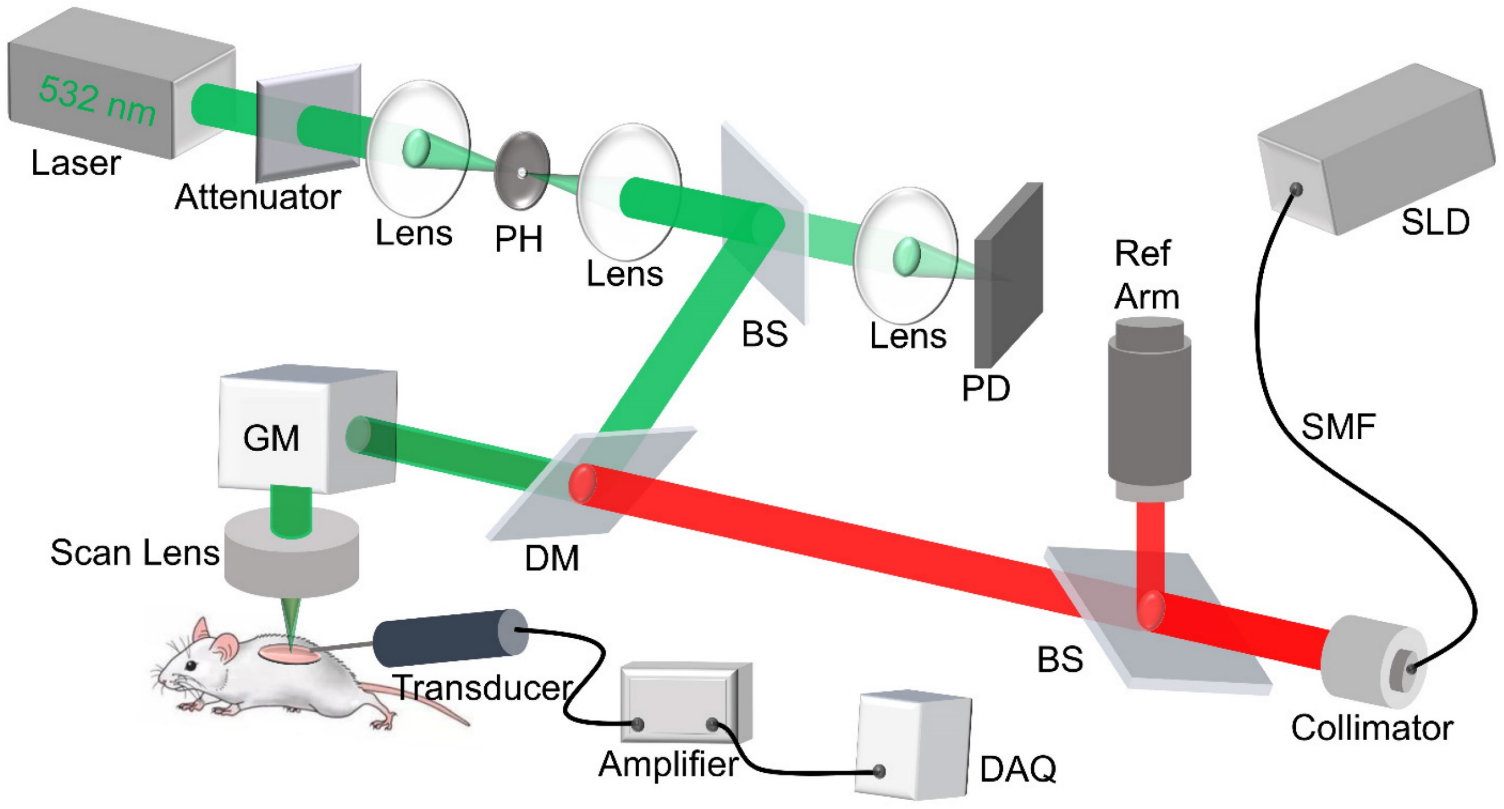
Schematic diagram of the dual modality imaging system, in which the green path shows the PAM light path, and the red path shows the OCT light path. PH: pinhole, DM: dichroic mirror, PD: photodiode, SMF: single mode fiber, SLD: super luminescent diode, GM: galvo mirror, DAQ: data acquisition.

The custom-built dual modality imaging system shown in Figure 1 offers high spatial resolution and fast image acquisition for non-invasive *in vivo* imaging. The green and the red paths indicate the PAM and the OCT working paths, respectively. They were integrated together through a dichroic mirror, and shared the same scanning path during the imaging. For PAM, a fiber laser (GLPM-16-1-10-M, IPG Photonics, Marlborough, MA, USA) working at 532 nm with a pulse repetition rate (PRR) set to 50 kHz was used as the light source. The laser was delivered to the biomaterial scaffold after beam shaping followed by passing through a scan lens and the imaging window. The induced photoacoustic (PA) waves were detected by a custom-built, needle-shaped ultrasonic transducer (Optosonic Inc., Arcadia, CA, USA) with a central frequency of 30 MHz and 60% bandwidth, which was coupled with the imaging window through gel drops (Systane, Alcon, TX, USA). The PA signal was amplified by applying a homemade low-noise preamplifier and collected by a 250 MHz digitizer (RazorMax PCIe CSE161G4, Dynamic Signal Inc, San Bruno, CA, USA). For OCT, a spectral domain OCT platform (TEL321, Thorlabs, Newton, NJ, USA) was integrated with the PAM to ensure coaxial illumination of both imaging modalities. For each imaging session, PAM was conducted first to assess vascular structure, followed by OCT to obtain scaffold structure. This system is an upgrade from our previous multi-modality imaging setup 29, 30, with a lateral resolution of 4 μm for both modalities as well as an axial resolution of 37.0 μm and 4 μm for PAM and OCT imaging modalities, respectively. A coverslip sample with coated chromium gratings (linewidth: 10 µm, pitch: 40 µm) providing excellent optical absorption and reflection contrasts as well as sharp edges was used as the target for lateral and axial resolution calibration of PAM and OCT. The lateral resolution was quantified by the fit of line spread function, and the axial resolution was calculated by the shift of a typical A-line signal. The quantified lateral resolutions of PAM and OCT were 4.1 μm and 3.8 μm, respectively; and the quantified axial resolutions of PAM and OCT were 37.0 μm and 4.0 μm, respectively. The imaging depths for PAM and OCT modality were 400 μm and 1 mm, respectively, validated by the tissue-mimic phantom and scaffold *in vitro*.

### Scaffold fabrication

Implantable polymer scaffolds were manufactured from poly(ε-caprolactone) (PCL) (Lactel). Briefly, PCL and sodium chloride were combined at a 1:30 mass ratio in a dual-shaft mixer heated to 85 ˚C to generate a polymer melt dispersion mixture. The melt dispersion mixture was transferred to a steel 5 mm die and pressed at 1,500 PSI for 30 s. The resulting discs were then annealed at 65 ˚C for 5 min per side on a hot plate prior to being salt leached in MilliQ water overnight to generate a disk with microporous architecture. The annealed and leached 5 mm scaffolds were then sterilized in ethanol for 60 s and rinsed twice with sterile water. Sterile scaffolds were then frozen at -80 ˚C for at least 5 h before subcutaneous implantation.

### Scaffold implantation

**Figure 2:**
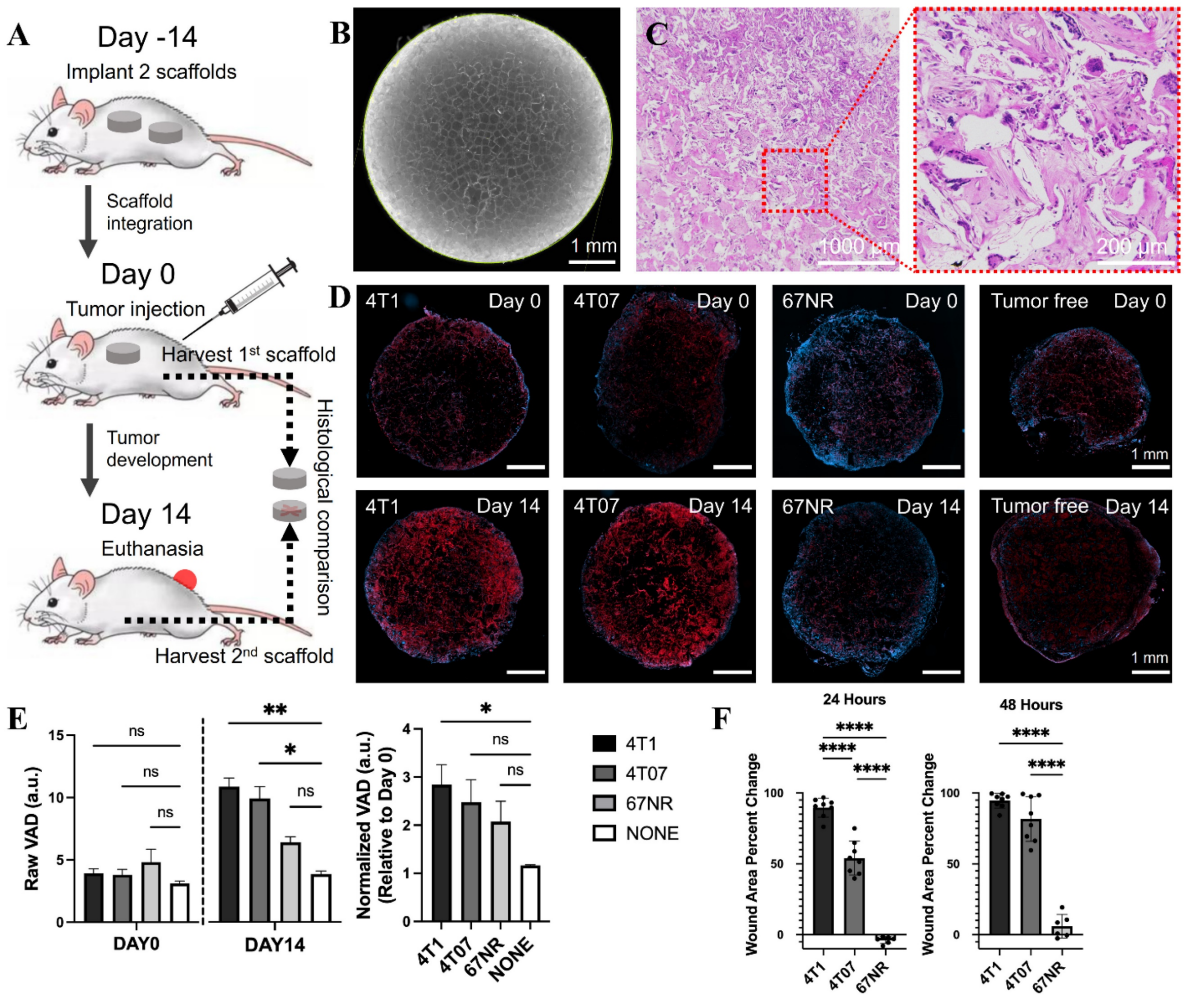
(A) Experimental timeline of the metastatic potential validation via vasculature analysis. (B) Microscopic and (C) H&E-stained images of the microporous scaffold. (D) Histological images of scaffolds from 4 groups, i.e., 4T1, 4T07, 67NR, and tumor free, at the beginning (day 0) and ending (day 14) time points, in which the red and blue colors indicate the vascular endothelial cells and DAPI, respectively. (E) Statistical analysis for the scaffolds with different cell lines (4T1, 4T07, and 67NR) as well as tumor free (control), including the raw data at day 0 and day 14 (left panel), and the normalized data (right panel). (F) Wound healing assay results for the three cell lines, represented as precent of wound closure over time.

**Figure 3:**
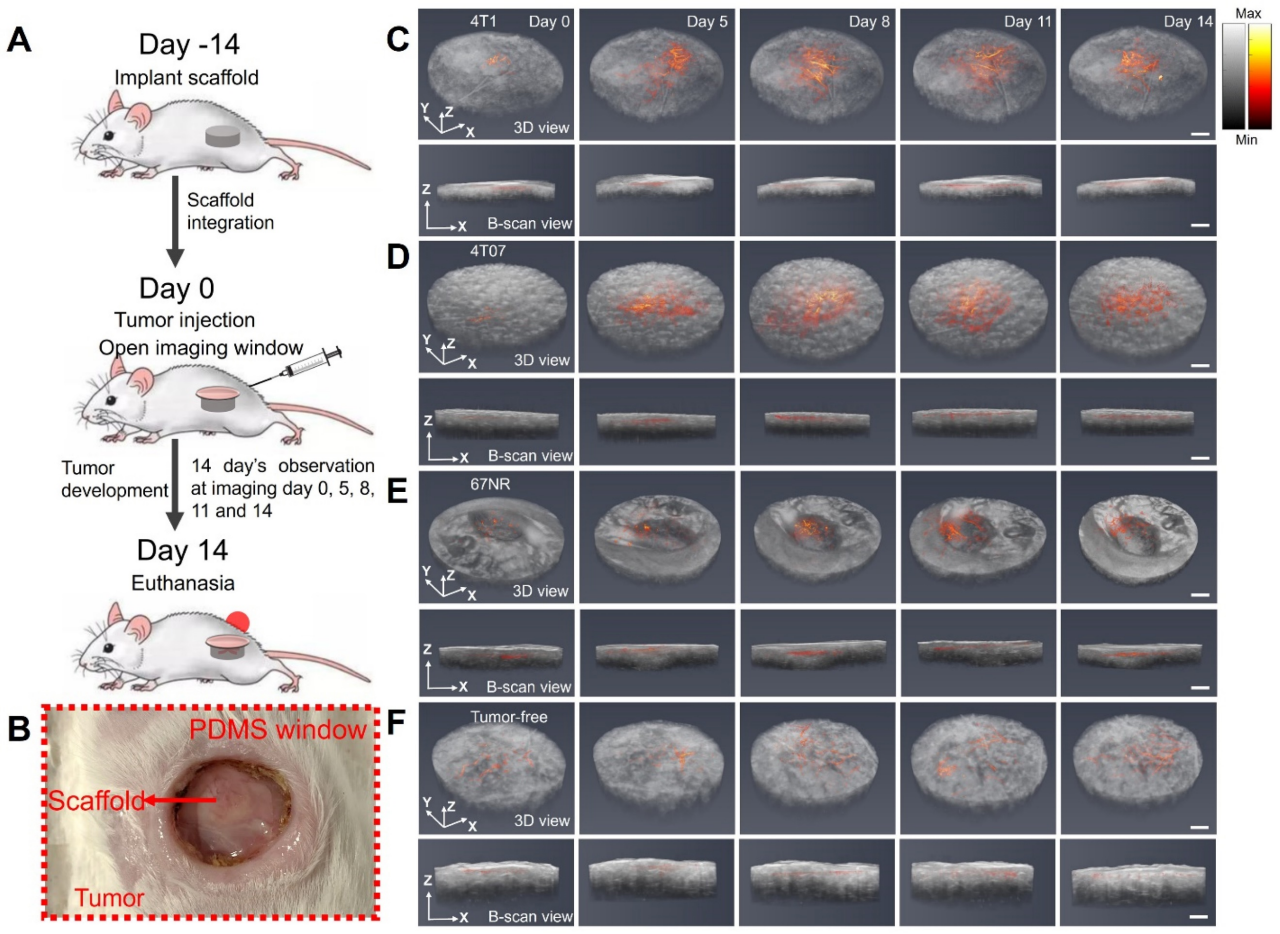
(A) Schematic diagram and experimental timeline of the *in vivo* imaging study. (B) Photograph of the PDMS window. (C-F) 3D PAM and OCT fusion images of selected scaffolds over 14 days, from 4T1 (C), 4T07 (D), 67NR (E), and tumor-free (F) groups, respectively. For each group, 3D rendering view and 2D B-scan results were presented. Scale bar: 1 mm.

Animal studies were performed in accordance with institutional guidelines and protocols approved by the University of Michigan Institutional Animal Care and Use Committee (PRO00012098: Multi-modality imaging of implanted scaffolds *in vivo*; PI: Xueding Wang). Eight-week-old female BALB/c mice from Jackson Laboratory were implanted with the scaffold subcutaneously. For histological validation, two scaffolds were implanted at day -14, and one scaffold was harvested at day 0 immediately prior to tumor inoculation as a baseline reference. The second scaffold was harvested at day 14 to compare vascular structure relative to the initial time point (**Figure 2A**). For the photoacoustic imaging group, one scaffold was implanted at day -14. Window implantation and tumor inoculation were both conducted on day 0 when the scaffold was fully integrated with surrounding tissues, followed by another 14 days of photoacoustic observation on imaging days 0, 5, 8, 11 and 14 (**Figure 3A**). For surgical implantation procedures, animals were anesthetized via isoflurane (2%, inhaled). Carprofen analgesia (5 mg/kg) was administered subcutaneously, and ophthalmic lubricant was applied to both eyes. The surgical area was shaved and sterilized thrice with betadine followed by alcohol. Following incision, subcutaneous pockets were created, into which sterilized scaffolds were inserted. The skin was then closed using sterile wound clips (Reflex 7 mm, Roboz Surgical Instrument Co.). 24 h post-surgery, mice received a second subcutaneous injection of carprofen analgesia.

### Histology

For histological validation, scaffolds were retrieved from BALB/c mice 14 days after orthotopic tumor inoculation. Tissues were flash frozen in isopentane on dry ice, and then embedded in Tissue Tek O.C.T. Compound (Sakura Finetek). Frozen tissues were cryosectioned transversely into 14 µm thick sections and placed on Superfrost Plus microscope slides (Fisher). Tissues sections were then fixed by 4% PFA and washed thrice with 1x TBS buffer (4.36 g Tris-HCL, 0.64 g Tris-Base, 8 g NaCl, 0.2 g KCl, 500 mL diH20). Subsequently, tissue sections were incubated with recombinant anti-GSL-1 primary antibody (VectorLabs, #DL-1208-.5, 1:100 dilution) in 1x TBS at room temperature for 2 h. Tissue sections were washed thrice with 1x TBS and mounted in DAPI Fluoromount-G (SouthernBiotech) before imaging (Zeiss Axio Observer).

### Wound healing assay

The three cell lines were cultured as stated in the tumor inoculation section. Cells were seeded at a density of 35k cells per well in a 96 well plate and allowed to reach confluency. The cell monolayer was wounded using a 200 µL pipette tip to create a scratch that is approximately 1 mm in width. The cells were washed 3 times with PBS and then the media was replaced with serum free media. Images of the wells were taken every 24 h for 48 h on a BioTek Cytation 5 imaging reader using the brightfield setting. The percentage wound closure was calculated relative to the original scratch area using the Wound-Healing-Size-Tool 31 ImageJ plugin.

### PDMS-based intravital imaging window

The PDMS silicon elastomer encapsulant kit (QSIL 216, PP&S), consisting of part A base and part B curing agent, was used to fabricate the imaging window. The base and curing agent were thoroughly mixed in a petri dish with a weight ratio of 10:1, and the mixture was put into a vacuum to degas the bubbles produced during the mixing procedure. A 24-well culture plate with 16 mm diameter hole was utilized as the mold, and appropriate amount of the mixture was dripped into the well. In this study, the desired thickness of the window was 100 μm, which was determined by the volume of the mixture dropped into the mold. Subsequently, the mold was placed in a 170 °F oven for 20 min to facilitate the curing of the mixture. Following this, the membrane was removed from the mold and baked at 180 °F for another 20 min to generate the imaging window 32. Those windows were washed by 75% alcohol and sterilized by autoclave procedure for surgery. As a linear (un-cross-linked) material, PDMS exhibits flexibility at the macroscopic level 33, ensuring that the window is stable with normal mouse movement. To preserve its integrity, the window was positioned in the dorsal region of the mouse, near its left hind leg such that it was beyond the reach of its paws. This position was chosen after testing several different locations. After surgery, each mouse was singly housed to prevent possible damage of the window caused by other mice. Before and after each imaging experiment, we gently cleaned the window surface with a rinsed cotton swab to maintain clarity.

### Imaging window implantation

Prior to the surgery, surgical table and all operative instruments underwent sterilization. The mouse was anesthetized with isoflurane (2%, inhaled) and placed in the supine position, with carprofen (5 mg/kg) injected for analgesia and a heating pad to maintain the body temperature. Ophthalmic lubricant was applied to both eyes. The surgical area was shaved and sterilized thrice with betadine and alcohol. An incision was then made above the scaffold, removing a circular area of skin to accommodate the PDMS window. The wound, especially the top surface of the scaffold, was thoroughly cleansed by sterile water to remove the coagulated blood. Then the PDMS-window was inserted into the circular wound, with edges covered by the skin. Once the window was in place and aligned, tissue adhesive (1469C, 3M Vetbond) was applied to bond the window to the skin. Finally, sterile saline was injected directly under the window to eliminate excess bubbles. Carprofen was administered postoperatively every 24 h to alleviate pain and distress of the mouse.

### Tumor inoculation

For our study, we utilized three murine TNBC cell lines: 67NR (nonmetastatic), 4T07 (micro-metastatic), and 4T1 (metastatic), all originating from a single tumor in a BALB/cfC3H female mouse. Female mice were selected, mirroring breast cancer's higher prevalence in women. The 67NR and 4T07 lines were acquired from the Karmanos Institute, Wayne State University, while the 4T1 line came from Perkin Elmer, MA. 4T07 and 67NR cells were cultured in high glucose DMEM, enriched with 10% fetal bovine serum, 2 mM L-glutamine, and 0.1 mM nonessential amino acids. 4T1 cells were cultured in RPMI with GlutaMAX, supplemented with 10% fetal bovine serum. Cells were thawed from storage in liquid nitrogen into their respective media which was changed every other day. After 5 days of culture, cells were collected and resuspended at a concentration of 40 million cells/mL in phosphate buffered saline (PBS) and stored on ice. Mice received 50 µL orthotopic inoculation of 67NR, 4T07, or 4T1 cells in the fourth, right mammary fat pad (2 million cells/mouse).

### *In vivo* longitudinal PAM and OCT imaging of scaffolds

Experimental mouse was anesthetized via isoflurane (2%, inhaled) and placed on a holder with the imaging window facing upwards. PAM and OCT imaging were carried out in accordance with the descriptions on Section 2.1. Imaging experiments were conducted after the window implantation, and the initial observation was assumed as imaging day 0, which was the same day of tumor inoculation, and the results obtained on that day were considered as baseline for further analysis. Subsequent longitudinal observations were performed on imaging days 5, 8, 11, and 14 to assess the vascularization within the scaffold. After 14-day imaging, the mice were euthanized, and the scaffolds were collected for histological analysis to verify the imaging results.

### 3D image fusion

PAM and OCT imaging results were coregistered to provide a comprehensive depiction within the scaffold through 3D image fusion. Due to coaxially aligned illumination lights for both imaging modalities, the XY planes for these results were coregistered by simply scaling the images size. Z-axial positions were also calculated based on the imaging parameter settings. The PAM image provided vascular structure within the scaffold, and the OCT image provided tomographic details of the scaffold. Here, AMIRA (Mercury Computer Systems, Berlin, Germany) was applied to complete the 3D fusion processes.

### Vasculature quantification

To simplify the analysis model, 2D PAM maximum intensity projection (MIP) images were utilized for vasculature quantification. An open-source software, OCTAVA 34, was applied. By importing PAM images into the software, several quantitative metrics for vasculature were extracted, including vessel area density (VAD), vessel mean tortuosity (VMT), total vessel length (TVL), and distribution of vessel diameter. Here, VAD is described as the perfused blood vessel area divided by the total image area, VMT is evaluated from the skeletonized image using the arc length-over-chord ratio, and TVL is the total length of all observable vessels measured along the vessel centerline 35. To eliminate subjects' variation, the metrics for different time points were normalized by the results of day 0. Following data normalization, those metrics from different subjects were grouped for further statistical analysis.

### Statistics

All statistical analyses were performed using GraphPad Prism software (GraphPad Software, Inc., La Jolla, CA). All bar graphs were plotted as the mean ± standard error of the mean (SEM) of measured quantities. The sample size for all the groups was n = 8 in this study. Statistically significant differences between groups were determined by using unpaired t-test, with differences denoted as * p < 0.05, ** p < 0.01, *** p < 0.001, **** p < 0.0001.

## Results

### Vasculature density and metastatic potential in TNBC models verified by histology and wound healing assay

We initially examined the relationship between vasculature density and metastasis within three TNBC models: 4T1, 4T07, and 67NR, each exemplifying a distinct stage of the metastatic cascade. Immunocompetent BALB/c mice were implanted with 2 microporous scaffolds (**Figure 2B**) (5-mm diameter, 2-mm in thick, 250-425 µm pores) composed of polycaprolactone (PCL) in the dorsal subcutaneous space. The scaffolds microporous architecture facilitates the colonization of cells throughout its thickness. After 2 weeks, one scaffold was collected for baseline reference, and the mice were orthotopically inoculated (day 0) with one of the triple-negative tumor cells (4T1, 4T07, or 67NR). The other scaffold was retrieved after another 14 days for histological analysis (**Figure 2A**). Vascular endothelial cells, positively related to vasculature density, were identified by expression of GSL-1 via immunofluorescence microscopy (the red-color area in **Figure 2D**), which indicated that at baseline (day 0) vasculature density was consistent across all models, including healthy controls.

By day 14 post-tumor inoculation, a marked variation in vasculature density was observed between the tumor models and the control (day 14 in **Figure 2D**). The highly metastatic 4T1 model exhibited the highest vasculature density. Conversely, the 67NR model, which does not invade beyond the primary tumor, had the lowest mean vasculature density. The 4T07 model, representing a micrometastatic model, displayed a density of vascular endothelial cells between the 4T1 and 67NR models. These findings were further substantiated when the day 14 vasculature densities were normalized to the baseline values (**Figure 2E**), revealing a clear gradation in line with the metastatic progression of the cancer models. This pattern supports our hypothesis that vasculature density is indicative of metastatic aptitude in breast cancer models.

A wound assay was also performed to compare the aggressiveness of the three cell lines. The percent change of the wound area (**Figure 2F**) indicated that the 4T1 cells were the most aggressive, followed by the 4T07 cells, followed by the 67NR cells that showed minimal wound closure. The results were consistent with the histological analysis and corroborated the findings of previous reports 28, 36, 37.

### 3D fusion images of vasculature and scaffold morphology captured by PAM and OCT

Subsequently, our objective was to longitudinally visualize changes in vasculature within the three tumor models (4T1, 4T07, and 67NR) through the window by using the dual-modality imaging. Scaffolds were initially implanted into the mouse, and an imaging window (**Figure 3B**) was opened 14 days later when the scaffold was fully integrated. Tumor inoculation was also conducted at the same day when opened the window. Utilizing PAM and OCT imaging, we imaged the scaffolds over a period of another 14 days at days 0, 5, 8, 11, and 14. The resultant 3D fusion images of scaffolds from both tumor-bearing (**Figure 3C-E**) and tumor-free (**Figure 3F**) mice demonstrate the system's ability to generate 3D representations, with vasculature highlighted in red and scaffold architecture in gray.

Limited by the optical absorption and scattering in the scaffold, the PAM imaging achieved a penetration depth of 400 μm inside the scaffold. The imaging revealed consistent biomaterial morphology across different tumor models and over time. Significantly, the imaging showed pronounced vascular remodeling in the tumor-bearing groups compared to the tumor-free control, with differences in vascular density emerging as early as day 5 or day 8, respectively. In summary, the dual modality imaging effectively delineated scaffold morphology and vasculature alterations.

### Quantitative and statistical analysis of vascularization

**Figure 4:**
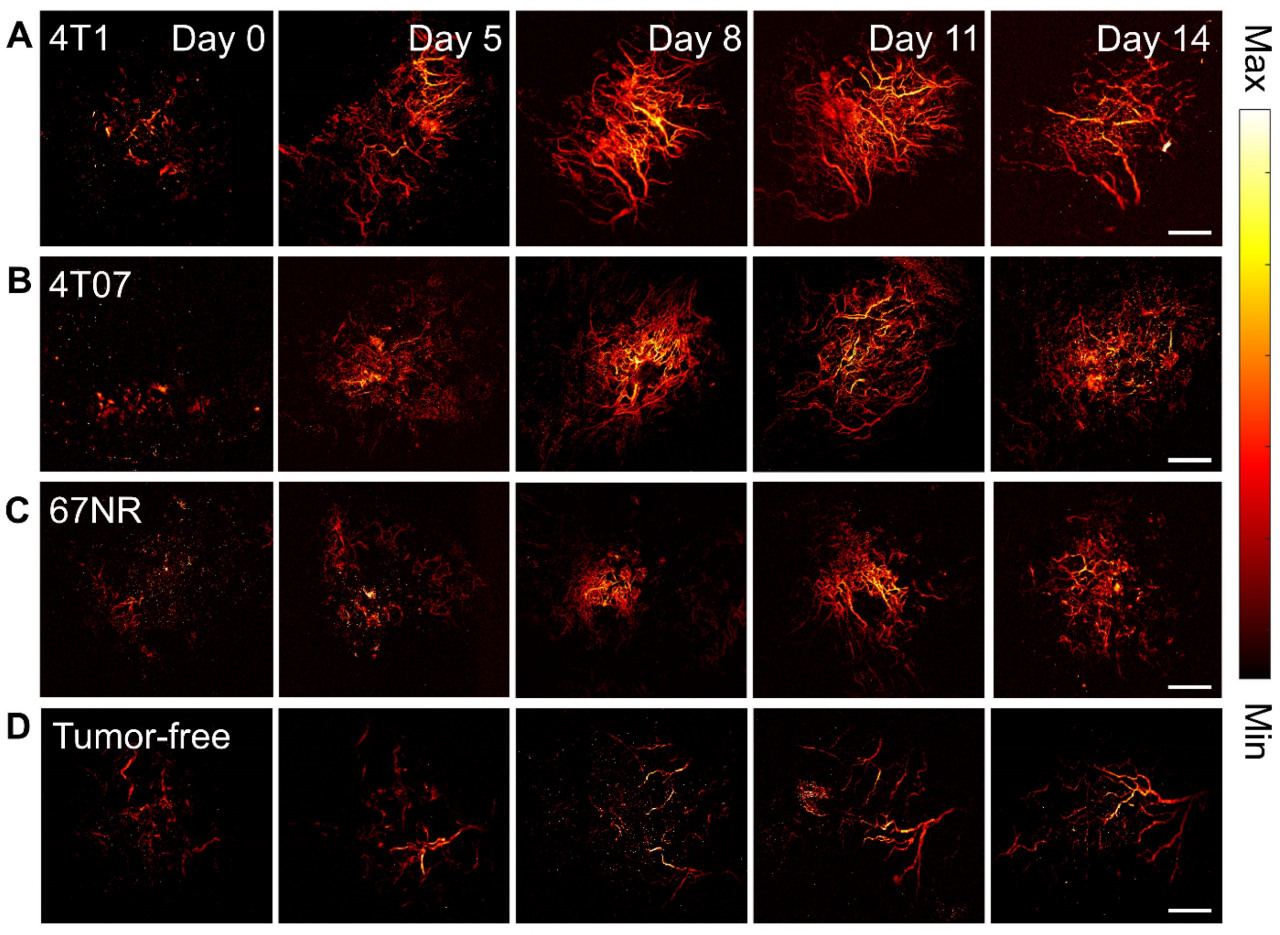
The comparison of PAM images (2D maximum projections) from 4T1 (A), 4T07 (B), 67NR (C), and tumor-free (D) groups over 14 days. Scale bar: 1 mm.

The vascular dynamics were quantified and correlated with the known dynamics of these models. We employed PAM imaging and initially, we transformed 3D PAM images into 2D maximum projections (**Figure 4**). This process elucidated the vascular remodeling in metastatic 4T1 and 4T07 models, contrasted with the stable vasculature observed in the 67NR and tumor-free mice. Employing OCTAVA, we quantified several vasculature metrics, which are pivotal for delineating vascular alterations over time. These metrics included vessel area density (VAD), which reflets the ratio of perfused blood vessels to the total imaged area; total vessel length (TVL), which represents the cumulative length of all vessels; vessel mean tortuosity (VMT), which indicates the complexity of vessel pathways; and the fitted distribution of vessel diameter. These metrics were selected for their strong correlation with angiogenesis progression. Studies have shown that as angiogenesis advances, both VAD and TVL increase, which reflects the growth and extension of new blood vessels within a tissue 38, 39. VMT, which measures the abnormal twisting of vessels is often seen in diseased vasculature and is a way to quantify uncontrolled vasculature 40. VMT thus measures the extent of diseased vasculature that grows from unregulated angiogenesis that is dysfunctional. The distribution of vessel diameters was included because unregulated growth leads to a shift toward smaller, underdeveloped vessels 41.

**Figure 5:**
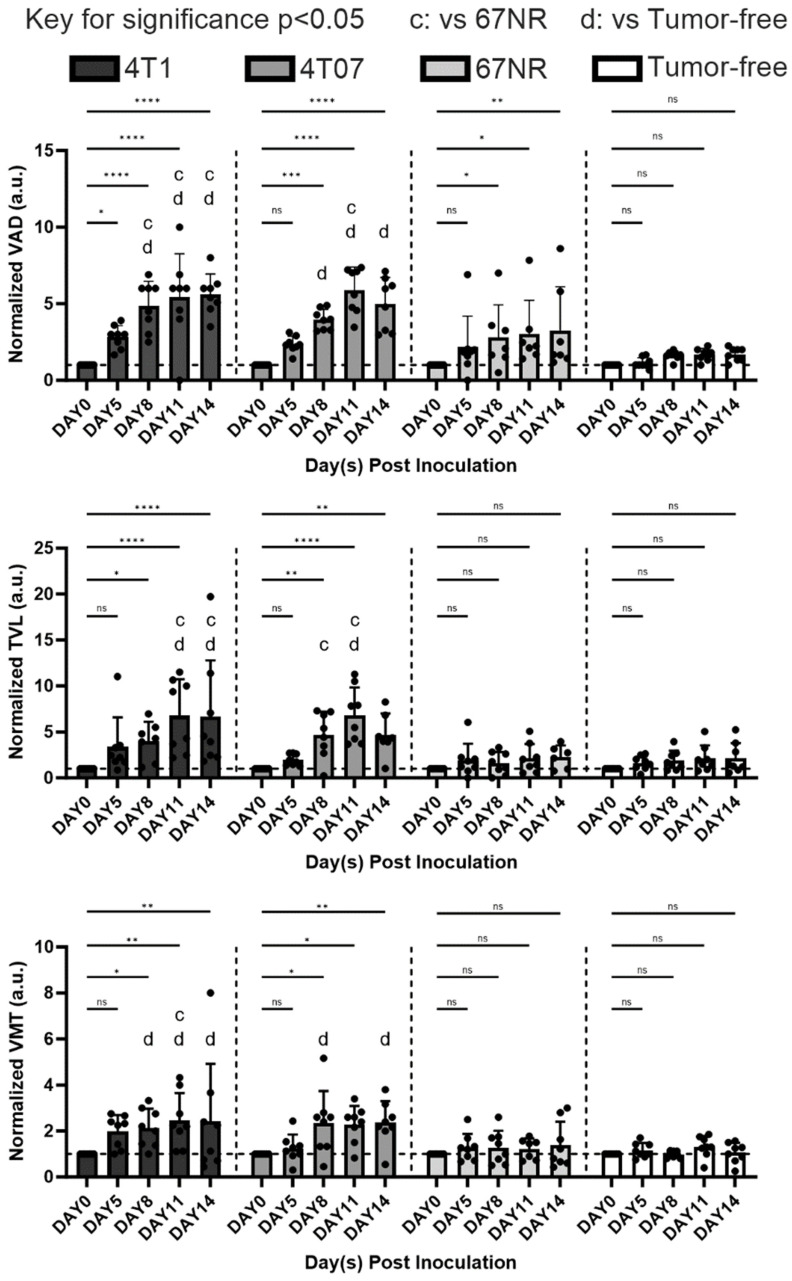
Quantitative analysis of different vasculature parameters, including normalized VAD, normalized TVL, and normalized VMT, over time post inoculation and between different groups (4T1, 4T07, 67NR, and tumor free). For all plots, error bars represent standard error of mean (SEM). n = 8 mice per condition. Statistically significant differences between groups were determined by using unpaired t-test, with differences denoted as * p < 0.05, ** p < 0.01, *** p < 0.001, and **** p < 0.0001 for comparing the measurements to baseline (day 0) via unpaired t test. The letters indicate significance p < 0.05, where “c” is significantly altered from 67NR and “d” from tumor-free via unpaired t test.

**Figure 6:**
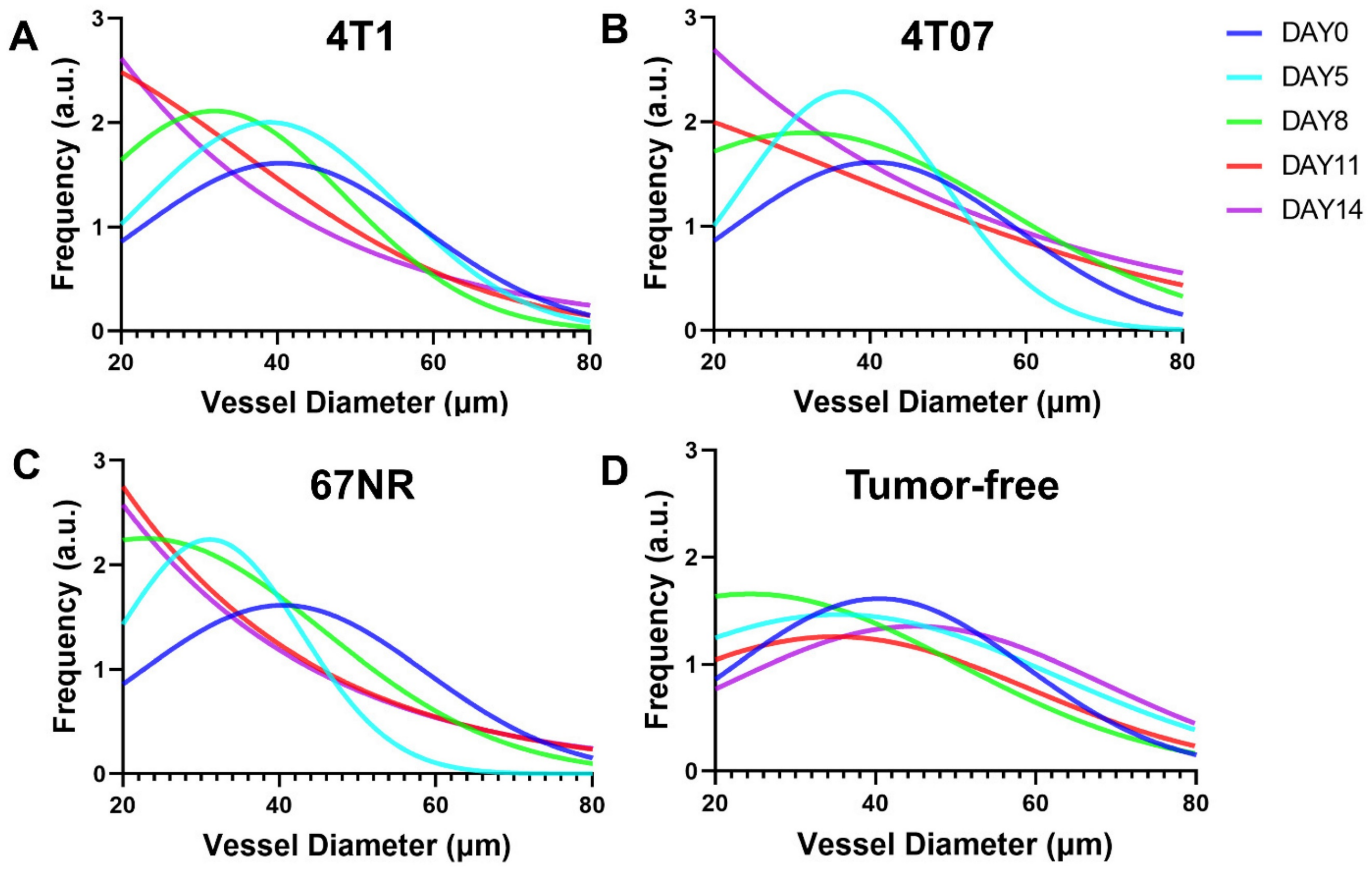
Vessel diameter distributions for (A) 4T1, (B) 4T07, (C) 67NR, and (D) tumor-free groups over 14 days. Obvious left shift of the distribution mean can be observed in the tumor-bearing groups but not in the tumor-free group, indicating more and more newly grown small vessels in the tumor-bearing group.

The analysis demonstrated that the 4T1 and 4T07 models have increases in VAD, TVL, and VMT over time. Significantly, these models showed elevated VAD, TVL, and VMT compared to tumor-free mice, becoming apparent by day 8 post inoculation. In contrast, the 67NR model displayed a slight increase in VAD over time; however, compared to tumor-free mice, no statistical significance was observed in any of the metrics (VAD, TVL, VMT) at any time point (**Figure 5**). Vasculature within tumor-bearing mice was observed to be more disorganized, suggesting that vascularization patterns may act as an early biomarker for metastatic activity. Additionally, vessel diameter distributions, analyzed through a Gaussian fit over 14 days (**Figure 6**), indicated consistent diameters in tumor-free mice. In comparison, tumor-bearing mice had a significant reduction in average vessel diameter over time, indicating more neovascularization.

## Discussion

Metastatic cancer is often detected due to patients self-reporting symptoms, with follow up imaging and biopsy used to identify the extent of disease. These approaches often identify advanced disease, which motivates the development of novel technologies for disease surveillance for early identification of disease. These technologies would allow for clinical intervention while the disease burden and heterogeneity are low. Herein, we applied a scaffold as a synthetic metastatic niche that provides a pre-defined site for analysis and investigated whether the vascular structures within the niche could indicate disease progression. Angiogenesis has been identified as a hallmark of cancer and is systemically altered in conjunction with disease progression 42. Biopsy has been the most common method for determining disease at the scaffold, yet we investigated the use of imaging technologies to assess the vascular network at the synthetic niche. Our imaging of the vascular network within the scaffold *in vivo*, longitudinally, provided the ability to detect disease and distinguish metastatic from non-invasive disease.

Quantitatively analyzing the vascular architecture of the tumor models demonstrated an increased vasculature as time progressed in the scaffolds of the aggressive models (4T1 and 4T07) relative to the non-metastatic model (67NR). Most research on correlating angiogenesis to disease aggressiveness focuses on taking measurements at the primary tumor. While intertumoral angiogenesis is important since it allows for tumor growth and cell intravasation, systemic angiogenesis allows for the establishment of secondary metastasis by establishing a premetastatic site to which tumor cells can extravasate. Intertumoral micro-vessel density (IMVD) has served as a clinical prognostic marker for survival in various cancers, including advanced breast cancer 43. Measured through immunohistochemistry of primary tumor biopsies for endothelial markers like CD31 44, IMVD quantifies angiogenesis at a specific point in time, despite the dynamic nature of this process. Even with its limitations, it is the sole method for tracking angiogenesis at the microscopic level in clinical practice. A correlation has been documented between high levels of IMVD, increased angiogenesis, and metastasis, which often results in poorer survival outcomes 19. *In vivo* imaging at the scaffold showed that in contrast with the 4T1 and 4T07 models, the 67NR model had less changes over time in vascular structures and were comparable to the tumor-free control mice (**Figure 5**). The VAD measurements correlated well with measurements obtained using the more traditional histological analysis but did not require a tissue biopsy. Scaffolds from the 4T1 and 4T07 models also exhibited an increase in vessel mean tortuosity (VMT) over time, and this tortuosity has been correlated with cancer aggressiveness 45. Collectively, these studies suggest a method for observing systemic angiogenesis at metastatic sites.

The natural absorption of hemoglobin at 532 nm makes PAM suitable for label-free vascular imaging, with high sensitivity, high spatial resolution, and 3D depth penetration. A prior study was conducted only for vascularization tracking at the primary tumor site by using PAM as the only imaging modality, lacking extra imaging modality to study the overall tumor morphology 46. By adding OCT as the secondary imaging modality, our study offers both vasculature and spatial morphology of the metastatic niche. However, one limitation of this study was the use of single-laser wavelength at 532 nm for generating the PA signal, which did not allow the measurement of blood oxygen saturation. For further improvement, multi-wavelength laser could be applied to achieve functional imaging and molecular imaging, presenting a more comprehensive depiction of the microenvironment within scaffold. Another limitation here was the OCT provided limited diagnostic information for TNBC due to the lack of quantitative analysis for OCT images. In future studies, we plan to longitudinally leverage OCT for additional quantitative analyses, such as measuring tissue scattering properties, layer thicknesses, and microstructural changes within the scaffold. These parameters could offer diagnostic information and help cross-validation with PAM results. For instance, tissue scattering properties change as angiogenesis progresses, and by tracking these changes with OCT, this parameter can be used to verify PAM results. Similarly, microstructural changes, which reflect the vasculature, could serve as a potential complement for validating PAM findings. By expanding the scope of OCT in further experiments, we aim to enhance its utility in tumor diagnosis and prediction, and better address the limitations noted in the current study.

In conclusion, we used a PAM and OCT combined dual-modality imaging platform to longitudinally monitor angiogenesis induced by TNBC, using tumor models representative of multiple aspects of the metastatic cascade. A chamber model with a flexible imaging window was developed to provide a durable and optically clear field of view for imaging a biomaterial scaffold acting as a metastatic niche. OCT enabled visualization of the scaffold morphology, and PAM detected the changes in vasculature over 14 days. The imaging results, which were consistent with a more traditional histological analysis, confirmed our hypothesis that angiogenesis at the scaffold is positively correlated with the metastatic potential of TNBC cell lines.

## Funding

This research is sponsored in part by grants including NIH R01EY034325, 1R01CA243916, R01CA250499, R01AR060350, and 1R01CA272940.
